# Real-time, acquisition parameter-free voxel-wise patient-specific Monte Carlo dose reconstruction in whole-body CT scanning using deep neural networks

**DOI:** 10.1007/s00330-023-09839-y

**Published:** 2023-06-27

**Authors:** Yazdan Salimi, Azadeh Akhavanallaf, Zahra Mansouri, Isaac Shiri, Habib Zaidi

**Affiliations:** 1 Division of Nuclear Medicine and Molecular Imaging, Geneva University Hospital, CH-1211 Geneva, Switzerland; 2 Geneva University Neurocenter, Geneva University, CH_1205 Geneva, Switzerland; 3 Department of Nuclear Medicine and Molecular Imaging, University of Groningen, University Medical Center Groningen, Groningen, Netherlands; 4 Department of Nuclear Medicine, University of Southern Denmark, DK-500 Odense, Denmark

**Keywords:** Computed tomography, Monte Carlo method, Deep learning, Neural networks

## Abstract

**Objective:**

We propose a deep learning-guided approach to generate voxel-based absorbed dose maps from whole-body CT acquisitions.

**Methods:**

The voxel-wise dose maps corresponding to each source position/angle were calculated using Monte Carlo (MC) simulations considering patient- and scanner-specific characteristics (SP_MC). The dose distribution in a uniform cylinder was computed through MC calculations (SP_uniform). The density map and SP_uniform dose maps were fed into a residual deep neural network (DNN) to predict SP_MC through an image regression task. The whole-body dose maps reconstructed by the DNN and MC were compared in the 11 test cases scanned with two tube voltages through transfer learning with/without tube current modulation (TCM). The voxel-wise and organ-wise dose evaluations, such as mean error (ME, mGy), mean absolute error (MAE, mGy), relative error (RE, %), and relative absolute error (RAE, %), were performed.

**Results:**

The model performance for the 120 kVp and TCM test set in terms of ME, MAE, RE, and RAE voxel-wise parameters was  − 0.0302 ± 0.0244 mGy, 0.0854 ± 0.0279 mGy,  − 1.13 ± 1.41%, and 7.17 ± 0.44%, respectively. The organ-wise errors for 120 kVp and TCM scenario averaged over all segmented organs in terms of ME, MAE, RE, and RAE were  − 0.144 ± 0.342 mGy, and 0.23 ± 0.28 mGy,  − 1.11 ± 2.90%, 2.34 ± 2.03%, respectively.

**Conclusion:**

Our proposed deep learning model is able to generate voxel-level dose maps from a whole-body CT scan with reasonable accuracy suitable for organ-level absorbed dose estimation.

**Clinical relevance statement:**

We proposed a novel method for voxel dose map calculation using deep neural networks. This work is clinically relevant since accurate dose calculation for patients can be carried out within acceptable computational time compared to lengthy Monte Carlo calculations.

**Key Points:**

*• We proposed a deep neural network approach as an alternative to Monte Carlo dose calculation.*

*• Our proposed deep learning model is able to generate voxel-level dose maps from a whole-body CT scan with reasonable accuracy, suitable for organ-level dose estimation.*

*• By generating a dose distribution from a single source position, our model can generate accurate and personalized dose maps for a wide range of acquisition parameters.*

**Supplementary information:**

The online version contains supplementary material available at 10.1007/s00330-023-09839-y.

## Introduction

The capability of visualizing inside the human body through non-invasive medical imaging examinations is a tremendous opportunity to diagnose various pathologies. X-ray computed tomography (CT) is one of the prevalent imaging modalities used in the initial clinical diagnosis, follow-up, staging, radiation therapy planning, and in emergency departments to provide valuable information for a wide range of indications [1]. In addition, CT is also commonly attached to nuclear medicine instrumentation, such as single-photon emission computed tomography (SPECT) or positron emission tomography (PET), for concurrent SPECT/CT [2] or PET/CT [3] imaging on hybrid imaging devices. At the same time, CT, one of the high-dose examinations, is responsible for a significant part of the ionizing radiation exposure of patients [4, 5]. The International Commission on Radiological Protection (ICRP) [6] suggested estimating the radiation dose delivered to patients from medical imaging procedures toward the optimization rule known as ALARA in order to minimize the risks through the appropriate use of ionizing radiation.

The recent emphasis on personalized medicine and patient-specific justification/optimization substantiates the critical demand to calculate specific parameters related to radiation risks [7–10]. The organ dose is a requirement for patient-specific dose calculation and has a good correlation with radiation risks [9]. On the other hand, it has been shown that the radiation dose delivered to specific organs can reach the deterministic dose levels, especially in serial CT examinations, which is common practice in patient follow-up, e.g., in the recent COVID-19 pandemic [11–13].

The estimation of organ doses can be performed using multiple methodologies. The most straightforward approach uses conversion factors specific to the scanning protocols. An alternative option is to use dedicated software tools, such as ImpactDose^1^ and Radimetrics [14]. Both above-mentioned software packages proved to have a low correlation with organ doses calculated by more accurate Monte Carlo (MC) simulation tools using patient-specific or reference computational models [15, 16], particularly when the tube current modulation (TCM) system is activated [17–19]. While MC calculations using patient-specific computational models is deemed to be the most accurate approach and is often regarded as the gold standard technique, its downsides, including computational time, high computational burden, and required expertise in computer programming, limit its adoption in clinical setting. Exploiting the parallel computational power of GPUs enabled MC calculations to be faster and more suited for adoption in clinical setting [20, 21]. Yet, the complexity associated with the technique remains a significant hurdle. Deep learning-based algorithms are currently used in various medical imaging applications, including image regression [22], registration [23], segmentation [24], radiation dosimetry calculation [25, 26], and optimization [27, 28]. This study aimed to develop a fully automated method to estimate patient-specific MC-based dose maps associated with whole-body (WB) CT examinations in real time using deep neural network algorithms.

## Materials and methods

### Study population

**Fig. 1:**
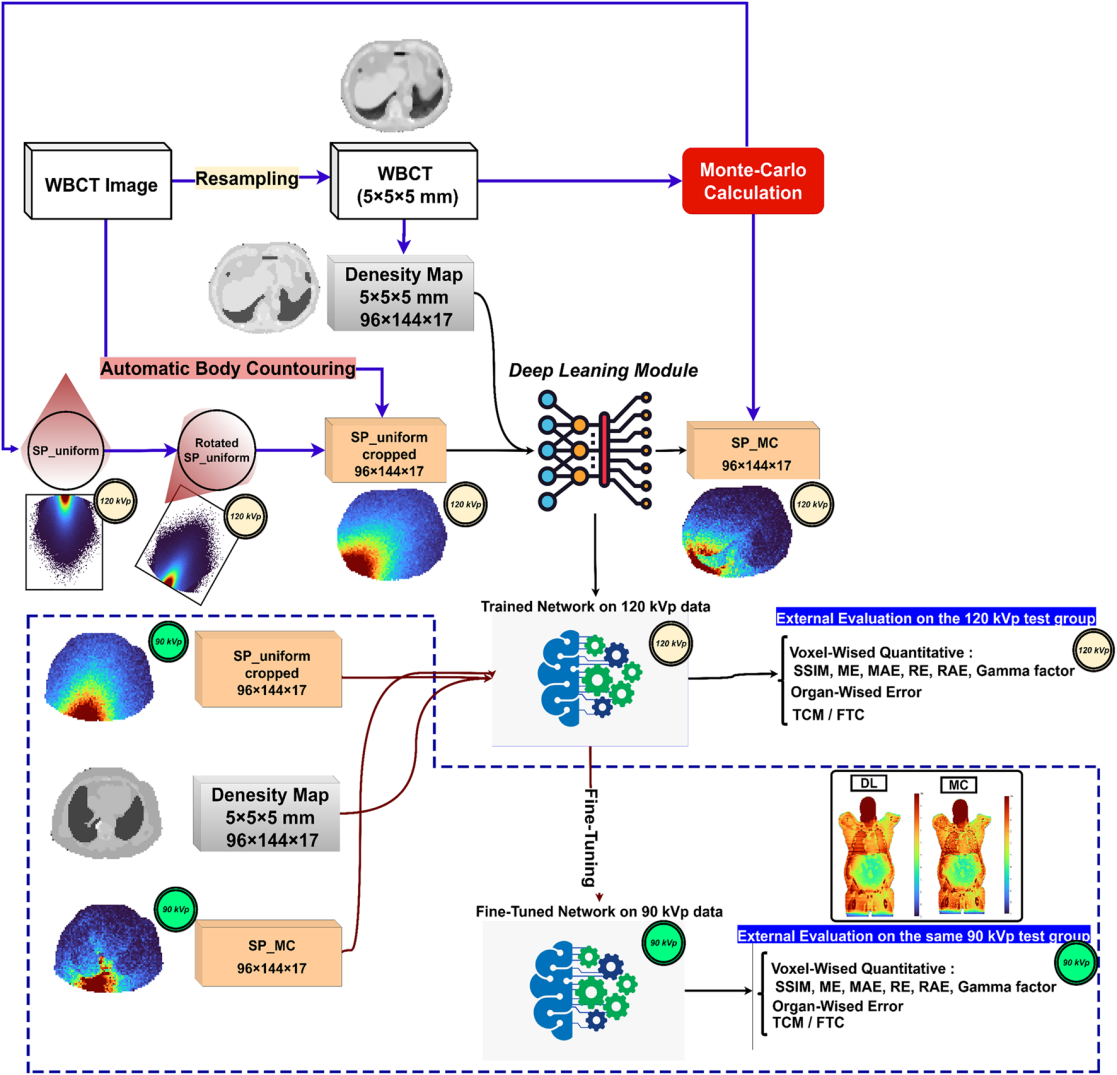
Flowchart summarizing the different steps involved in the implementation of the whole process. The blue dashed line shows the 90 kVp generalizability test. DL: Deep learning. MC: Monte Carlo

This study included 63 patients (35 male and 28 females) who underwent whole-body PET/CT imaging on a Biograph mCT scanner (Siemens Healthineers). All CT scans were performed in helical mode using 120 kVp tube potential, and Siemens CareDose4D TCM was activated. Images were reconstructed with the extended 70 cm field-of-view option, voxel size equal to 1.523 mm in the axial plane, and 1.4 mm slice thickness using a filtered-back projection algorithm. Figure 1 shows the flowchart of the different steps followed in this study protocol.

### Monte Carlo simulations

CT HU values were converted to density maps using linear multi-regression models for the segmentation of CT images into different tissue densities, as proposed by Schneider et al [29]. Subsequently, the resulting density maps were resampled to 5 mm^3^ cubic isotropic voxel size. The essential components incorporated into MC simulations, including accurate source model and protocol-related parameters, were adopted from our in-house MC simulation code developed and validated in a previous study [30]. The acquisition parameters, including tube voltage, collimation width, table speed, rotation time, pitch, and tube current modulation, were implemented in this simulation. This simulator is based on the MCNPX general-purpose Monte Carlo radiation transport code (version 2.6) [31].

The output of MC simulations is a 3D dose map for a single source position (SP_MC) with limited axial coverage. Monte Carlo simulations were run for multiple discrete source positions to simulate helical whole-body CT scanning. A random starting location was generated for the source owing to the lack of information about the tube start angle in the DICOM header. Accordingly, a spiral motion of the source position in 2 mm axial intervals along the Z-axis (craniocaudal axis) was modeled. Finally, considering the longitudinal tube current modulation (extracted from the DICOM header for TCM), simulated dose maps for each source position were multiplied by the corresponding tube current and were superimposed to construct the complete voxel dose distribution.

### Data preparation

MC calculations were performed for a total number of 63 patients with 120 kVp tube voltage. Then, by keeping all parameters similar, except kVp, MC calculations were repeated with 90 kVp tube voltage for patients in the test group (11 cases) plus 20 cases randomly selected from the train and validation groups to perform the fine-tuning process described later in the text. The cases from the train and validation were used for performing transfer learning and fine-tuning.

### Monte Carlo calculation of radiation dose in a uniform cylinder at 90 and 120 kVp

A uniform water-filled cylinder with a 715 mm diameter located within the CT gantry was simulated, and the dose map for a single source position (zero degrees, located at the anterior point) was calculated for a large number of simulated events (4 × 10^10^ particles) tracked by the MC simulator. This dose map, referred to as the single-source position uniform map (SP_uniform), was calculated for two tube voltages, namely 90 and 120 kVp for a single source. It should be mentioned that the 90 kVp uniform dose maps were used for testing the network generalizability through fine-tuning.

### Generation of single-source position images and corresponding density maps

**Fig. 2:**
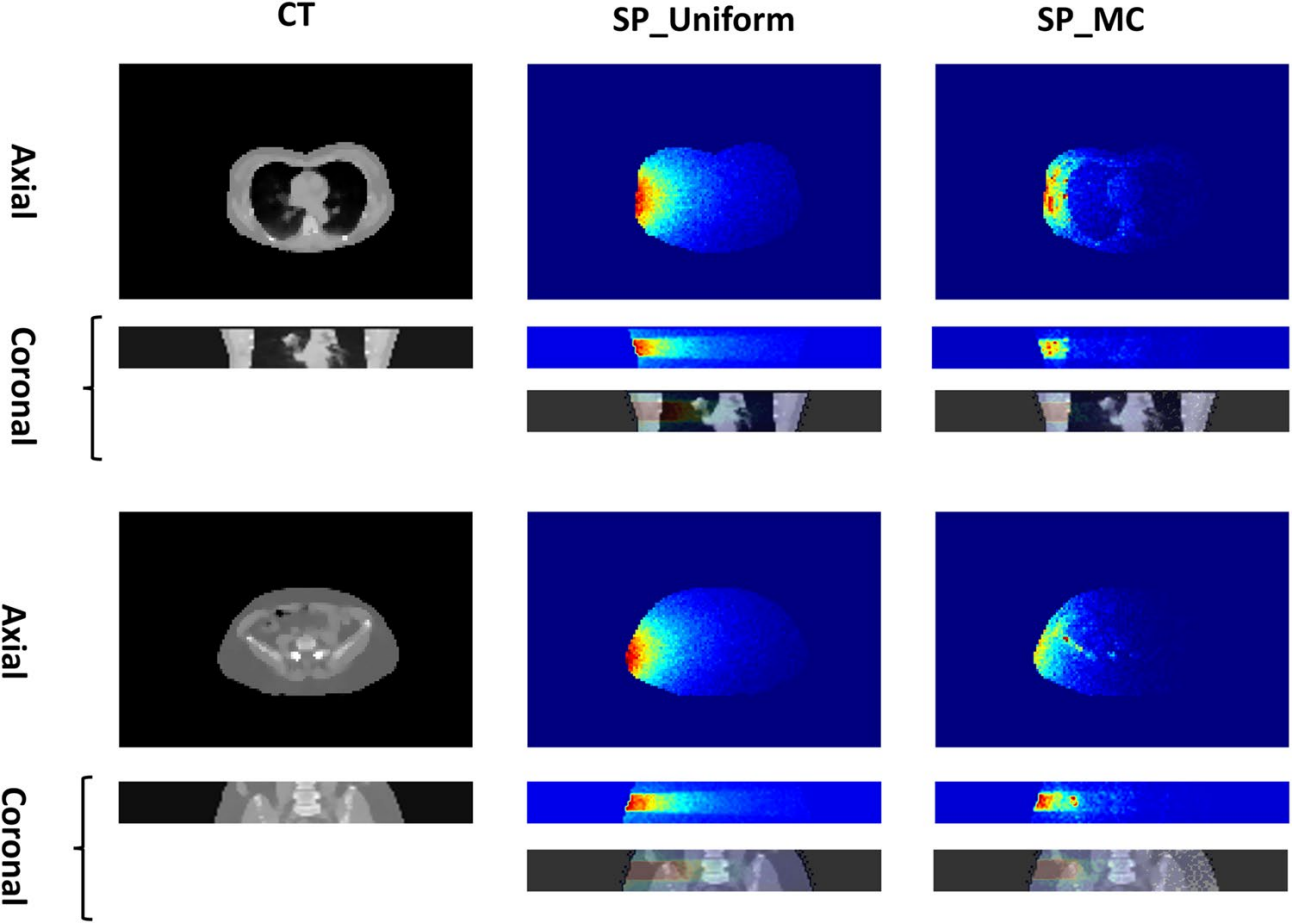
Examples of axial and coronal slices of CT, SP_Uniform, and SP_MC dose maps corresponding to a single source position/angle. In these cases, the X-ray tube is in the right lateral position

The body contour was automatically segmented on all CT images utilizing analytical image processing methods. All body contour segmentations were reviewed and confirmed visually. The MC output images (SP_MC) having a size of 96 × 144 × 17 voxels were saved, and the density map for the same axial coverage range was cropped to the same size. The SP_uniform images were cropped to the same axial coverage body contour and normalized to a conversion factor (CF) calculated by Eq. (1) to compensate for the effect of attenuation taking place in the SP_uniform dose calculation on the large cylinder. The SP-uniform dose image calculated for the source at zero-degree position (patients’ anterior in supine position) was rotated to match the desired angle (rotated SP_uniform in Fig. 2 and Supplementary Fig. 1).

1 $$\mathrm{CF }={e}^{\left({d}_{SP\_MC} - {d}_{SP\_uniform}\right)}.$$

where *e* is Euler's number, ${d}_{SP\_MC}$ is the distance from the edge of the body contour to the X-ray tube source in a specific source position. ${d}_{SP\_uniform}$ is the distance from the edge of a large cylinder simulated to the source in a specific position. Since the cylinder size was larger than the size of our largest patient, the CF was always greater than 1. Figure 2 shows the examples of SP_MC, SP_Uniform, and the corresponding CT slices when the source is in the right lateral position.

The two images of SP_uniform and SP_MC were normalized by all voxel intensities by a fixed value. Each source position was saved in a separate image and used for training the neural network.

### Network architecture and training details

From all 63 WB CT images (27,632 source positions), 11 cases (4792 source positions) were used as the untouched test set. Figure 1 shows the steps performed in this study and examples of mentioned three images of SP_uniform, SP_MC, and density maps. The SP_unifrom in a unique source position/angle and the density map images were fed as input to the neural network to predict the SP_MC image as the output in the corresponding source position/angle. A deep residual network (ResNet) was trained in Python (TensorFlow) to generate the SP_MC images from the two mentioned inputs. The ResNet is composed of 20 convolutional layers (19 layers with kernel size 3 × 3 × 3 and the last layer with kernel size 1 × 1 × 1) where the image size is kept constant through the different layers (no down or up pooling was applied). Different feature levels, including low, medium, and high, were extracted by using dilation of 0 (first seven layers), 2 (six middle layers), and 4 (six last layers), respectively, in a convolutional kernel. Every two layers were connected using a residual connection to avoid gradient vanishing or exploding. The training was continued for 100 epochs using the “Adam” optimizer and L2 loss function. The initial learning rate of ${10}^{-3}$ was reduced in a piecewise method every five epochs. The trained network was tested on the external group datasets, and the deep neural network output was named SP_DL.

### Generalizability evaluation on 90 kVp data (fine-tuning)

To test the generalizability of the proposed model for kVps other than 120 kVp, we performed MC simulations to calculate the voxel dose maps by considering the 90 kVp spectrum on the untouched test group (11 cases) and 20 patients selected from the train and validation group. The same pre-processing steps mentioned earlier were followed to derive SP_MC, SP_uniform, and density maps at 90 kVp. According to AAPM report #148 and McCollough et al [32] study, we used the same density maps for training the network on 90 kVp images. The model trained on the 120 kVp dataset was stored. This model with the trained weight and biases was used to initialize the new training (fine-tuning) process through transfer learning. The fine-tuning process was continued for 50 epochs with 1e-7 learning rate and the weights and biases were updated according to 90 kVp datasets with SP_unifrom and density maps as input and SP_MC image as output to the model. SP_uniform and density maps were fed to the fine-tuned network on 90 kVp training datasets, and SP_DL images at 90 kVp were generated for the same test group (11 cases). These SP_DL images were compared to SP_MC images at 90 kVp.

### Dose map reconstruction from single source positions

The dose maps from the single source position were corrected by factors related to the tube calibration described in a previous study [30]. The tube current was extracted from the DICOM header. Then, the dose maps corresponding to a single source position/angle were superimposed to reconstruct the whole-body dose maps (WBDM) using both SP_MC and SP_DL dose maps, referred to as WBDM_MC and WBDM_DL, respectively. The final WBDM was a matrix of 96 × 144 × Z voxels, where Z is the image size along the Z-axis, and the voxel value is the absorbed dose in that voxel in units of milli-gray (mGy). We have considered two strategies for WBDM calculation, fixed 100 mA tube current (FTC) and TCM activated according to the actual tube current recorded from the DICOM images.

### Evaluation metrics

#### Voxel-wise quantitative dose evaluation

The WBDM_DL images were compared with WBDM_MC images serving as the standard of reference (ground truth) at the voxel level. Voxel-wise parameters, including structural similarity index (SSIM), peak signal-to-noise ratio (PSNR) mean error (ME, mGy), mean absolute error (MAE, mGy), relative error (RE, %), relative absolute error (RAE, %), and gamma pass rate, were calculated. Gamma pass rate, as described earlier by Low et al [33] with 1 mm and 1% criterion, was considered.

#### Organ-level dose evaluation

In addition to voxel-wise errors, eight organs, including the Liver, Heart, Bones, Kidneys (both), Spleen, Bladder, Lungs (both), and brain, were segmented manually on the test WBCT images. The organ doses were estimated by calculating the mean voxel value on WBDM images inside the organ segmentations. The organ absorbed doses calculated on WBDM_DL and WBDM_MC were compared for each organ in terms of mean error (ME, mGy), mean absolute error (MAE, mGy), relative error (RE, %), and relative absolute error (RAE, %). These voxel-wise and organ-wised metrics were calculated for both 90 kVp and 120 kVp external datasets, considering both FTC and TCM scenarios.

#### Statistical analysis

The Kolmogorov-Smirnov test was used to check the normality of distributions. The mentioned organ-wise evaluation metrics were compared between the two groups of 90 and 120 kVps using the Mann-Whitney test. Due to the small sample size in the test group (12 cases), we preferred not to perform statistical analysis to compare voxel-wise metrics between 90 and 120 kVp datasets. *p* values less than 0.05 were considered statistically significant.

## Results

### Patients demographics

The age of included patients was 58.9 ± 17.2 years. The average patients’ water equivalent diameter was 26.6 ± 2.7 (range 16.45–32.95) cm. The average tube current implemented by TCM was 140.7 ± 48.71 (56 to 306) mA. Table 1 summarizes the demographic information of patients.

**Table 1:** Demographic description of the test, train, and validation groups

Metric	Train and validation	Test
sex	29 male, 23 female	6 male, 5 female
Age (years)	60.1 ± 16.9	53.2 ± 17.9
kVp (KV)	120	120
Pitch	0.8	0.8
CTDIvol (mGy)	5.74 ± 2.70	8.33 ± 4.00
Patient height (cm)	169 ± 12	167 ± 12
Patient weight (Kg)	75.1 ± 15.8	76.4 ± 17.4
Tube current (mA)	135.5 ± 45.4	167.6 ± 67.8

### Voxel-wise error metrics

Table 2 summarizes the results of voxel-wise metrics for two external validation groups acquired at 90 and 120 kVp. The model performance for the 120 kVp and TCM test set in terms of voxel-wise parameters, including SSIM, PSNR, Gamma pass rate, ME, MAE, RE, and RAE, was 0.997 ± 0.002, 46.69 ± 1.98, 98.47 ± 0.81,  − 0.0359 ± 0.0244 mGy, 0.1091 ± 0.0279 mGy,  − 1.16 ± 1.41%, and 7.13 ± 0.44%, respectively. All voxel-wise parameters were in the same range for 120 kVp, TCM, and FTC test sets. The voxel-wise evaluation results after performing transfer learning and fine-tuning on 90 kVp data were also comparable to 120 kVp, except RAE, which was almost 1.5% higher in the 90 kVp test group compared with 120 kVp results (8.63 vs. 7.17). Considering the FTC and TCM scenarios, the performance of our model was almost similar in the 90 kVp test set.

**Table 2:** Summary of voxel-wise evaluation metrics at 90 and 120 kVp

	120 kVp		90 kVp	
	FTC	TCM	FTC	TCM
SSIM	0.997 ± 0.002 (0.993–0.998)	0.997 ± 0.002 (0.993–0.998)	0.994 ± 0.005 (0.981–0.998)	0.994 ± 0.005 (0.981–0.998)
PSNR	46.69 ± 1.98 (44.95–50.17)	47.68 ± 1.98 (44.95–50.17)	45.11 ± 3.85 (37.51–48.77)	46.18 ± 5.08 (37.48–51.66)
Gamma value	98.47 ± 0.81 (96.73–99.72)	98.91 ± 0.81 (96.73–99.72)	98.26 ± 1.29 (95.28–99.08)	98.64 ± 1.41 (95.28–99.68)
ME (mGy)	− 0.0359 ± 0.0244 (− 0.0826–0.0025)	− 0.0302 ± 0.0244 (− 0.0826–0.0025)	− 0.0167 ± 0.0149 (− 0.0372–0.0161)	− 0.0126 ± 0.0124 (0.0326–0.0133)
MAE (mGy)	0.1091 ± 0.0279 (0.0513–0.1401)	0.0854 ± 0.0279 (0.0513–0.1401)	0.1088 ± 0.0308 (0.0776–0.1626)	0.0892 ± 0.0462 (0.0471–0.1713)
RE (%)	− 1.16 ± 1.41 (− 3.72–1.39)	− 1.13 ± 1.41 (− 3.72–1.39)	0.27 ± 1.33 (− 1.99–2.00)	0.28 ± 1.33 (− 2.00–1.98)
RAE (%)	7.13 ± 0.44 (6.57–7.89)	7.17 ± 0.44 (6.57–7.89)	8.58 ± 1.83 (6.15–10.80)	8.63 ± 1.82 (6.19–10.82)

**Fig. 3:**
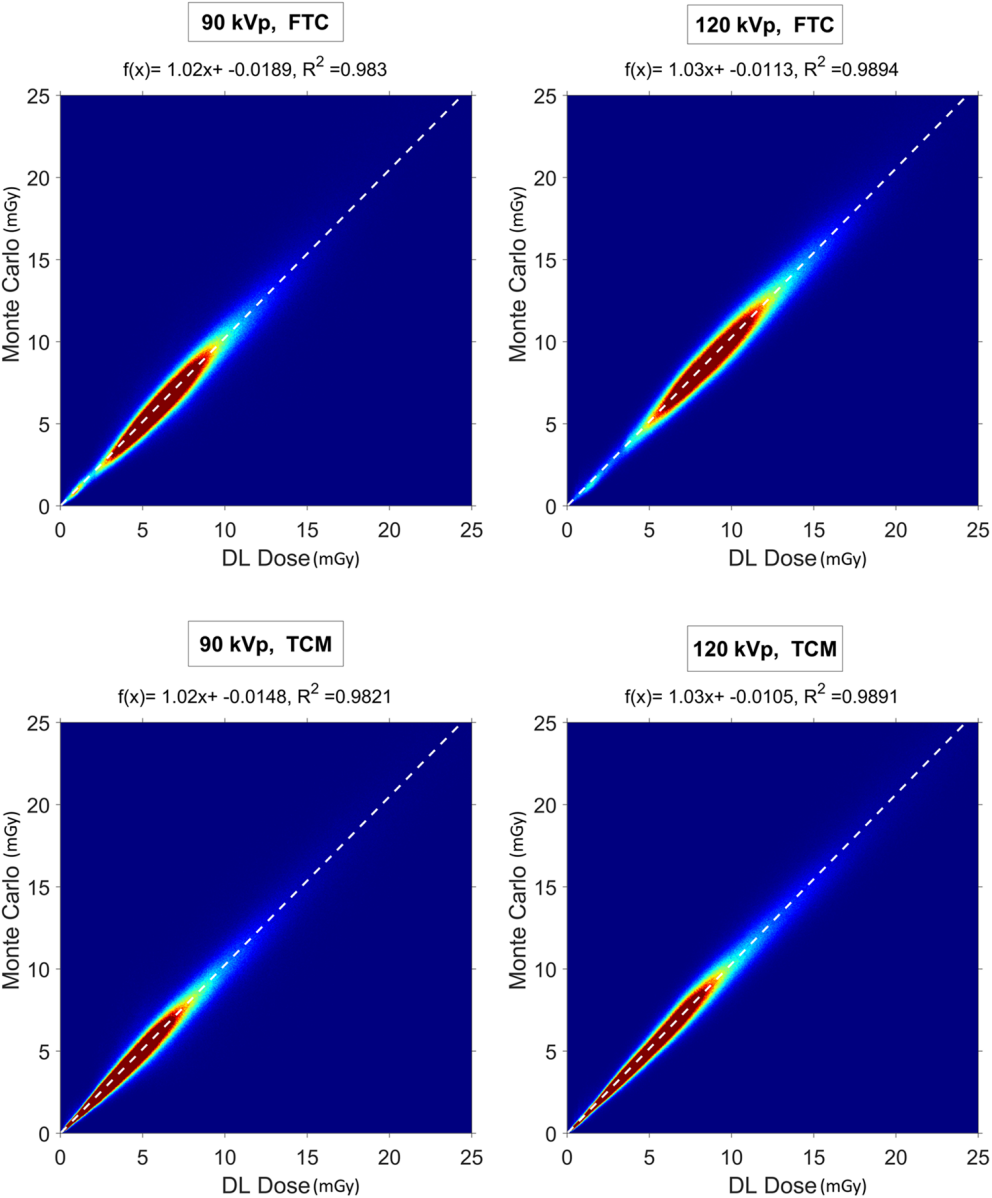
Joint-histograms comparing the voxel-wise doses of WBDM_DL and WBDM_MC at 90 kVp, FTC (top left), 120 kVp, FTC (top right), 90 kVp, TCM (bottom left), and 120 kVp, TCM (bottom right). The white dashed line shows the fitted line and the regression line equation. The correlation coefficient (*R*^2^) is also shown for each histogram

**Fig. 4:**
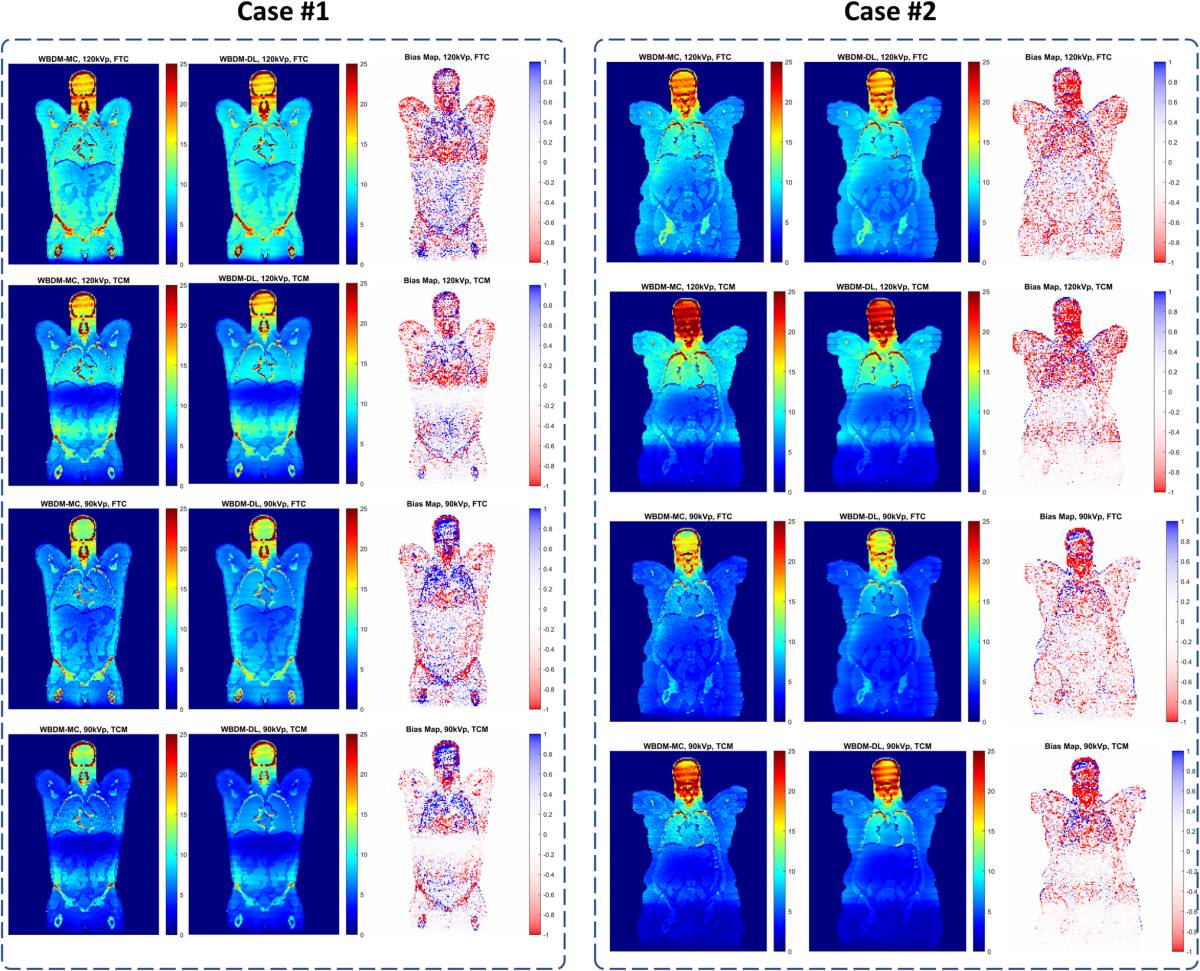
Coronal slices of WBDM_DL, WBDM_MC and the corresponding bias maps for two cases from the external test sets. The color bar unit in all images is mGy. The bias map was calculated as WBDM_DL – WBDM_MC, where blue pixels reflect positive bias map and the red pixels reflect negative bias map. The caption for each study displays the kVp and tube current scenario (TCM or FTC). Case #1: 74 y/o male, patient height = 172 cm, patient weight = 85 kg, average water equivalent diameter [34]  = 28.4 cm, the effective diameter at the largest slice = 32.9 cm. case #2: 65 y/o female, patient height = 158 cm, patient weight = 87 kg, water equivalent diameter = 29.5 cm, the effective diameter at the largest slice = 35.6 cm. The voxel value here is mGy, and the color bar is shown beside each image

Figure 3 shows the joint histogram comparing WBDM_DL and WBDM_MC doses at the voxel level. The high correlation depicted in Fig. 3 (*R*^2^ > 0.98) and other voxel-wise metrics show excellent agreement between MC and DL dose maps. Figure 4 shows two examples of WBDM_DL and WBDM_MC and their corresponding bias maps displayed in a coronal view for a combination of two kVps (90 and 120) and two TCM and FTC scenarios. The bias map shows excellent agreement between MC and DL results. The highest differences in terms of RAE (%) are depicted in the lung/chest wall interval and soft tissue/skull (bony tissue), where there is a gradient in density and chemical composition characteristics of biological tissues and, consequently, radiation interaction properties with tissues. The average RAE for all organs was always less than 4.5% for both kVps and TCM and FTC scenarios.

### Organ-wise error metrics

**Fig. 5:**
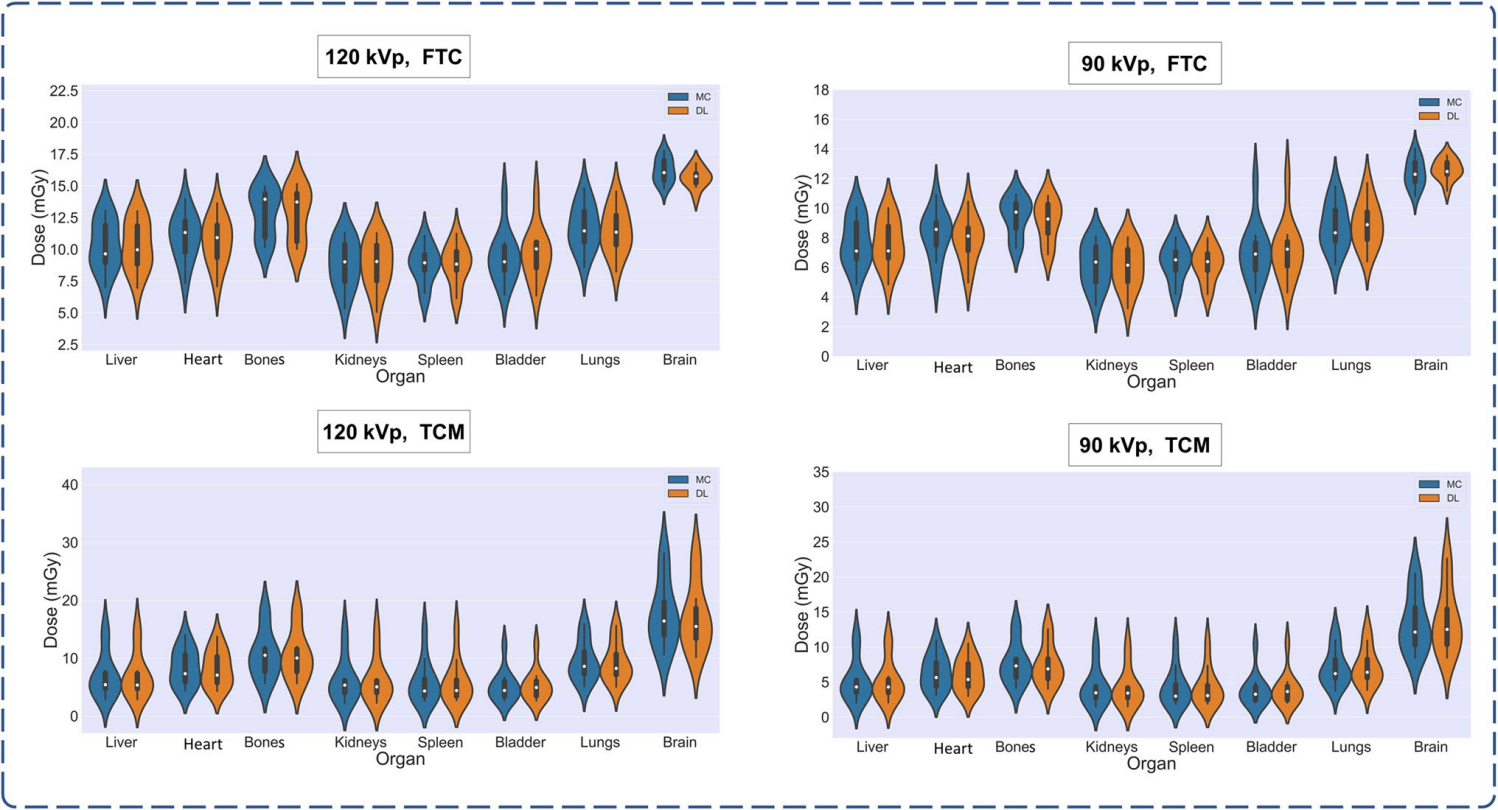
Violin plots of organ dose distributions calculated by MC (blue) and DL (orange) at 90 and 120 kVp for both FTC and TCM scenarios

**Fig. 6:**
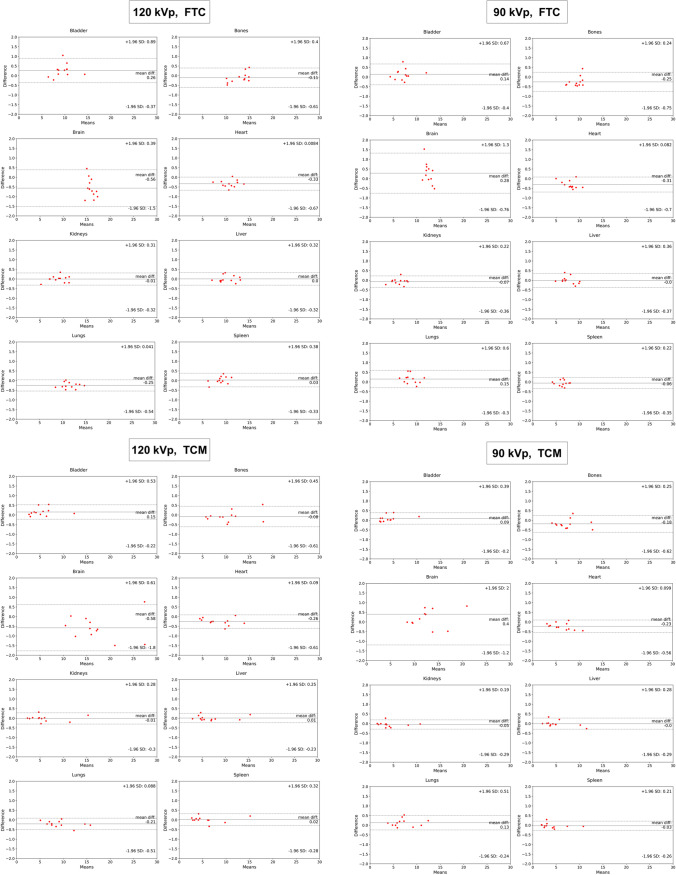
Bland-Altman plots comparing organ absorbed doses estimated by Monte Carlo and deep learning models

The organ-wise error for 120 kVp and TCM scenario averaged over all segmented organs in terms of RE (%), RAE (%), ME (mGy), and MAE (mGy) was  − 1.11 ± 2.90, 2.34 ± 2.03,  − 0.144 ± 0.342, and 0.23 ± 0.28 respectively. Tables 3 and 4 summarize organ-wise metrics calculated on 120 kVp and 90 kVp test sets, respectively. There was no statistically significant difference between the metrics calculated in organ-wise evaluations between the FTC and TCM performance in either the 90 and 120 kVp test sets (Mann-Whitney, *p* > 0.05). The highest average errors were observed in the heart, bones, and brain regions, where there is a higher gradient in density and surrounding tissues. Figure 5 compares organ doses measured on DL and MC reconstructed dose maps. The violin plots of organ doses, voxel-wise comparison of results, and gamma pass rate results show overall excellent agreement between the distributions of DL and MC organ doses. Figure 6 shows the Bland-Altman of RE and RAE (%) between the calculated organ doses.

**Table 3:** Outcome of organ-wise dose estimates for the 120 kVp dataset

		FTC				TCM			
	*Organ*	RE %	RAE %	ME (mGy)	MAE (mGy)	RE %	RAE %	ME (mGy)	MAE (mGy)
120 kVp	Liver	− 0.27 ± 1.54 (− 2–3.32)	1.26 ± 0.84 (0.42–3.32)	− 0.023 ± 0.158 (− 0.244–0.32)	0.128 ± 0.086 (0.055–0.32)	− 0.27 ± 1.54 (− 2–3.32)	1.26 ± 0.84 (0.42–3.32)	− 0.023 ± 0.158 (− 0.244–0.32)	0.128 ± 0.086 (0.055–0.32)
	Heart	− 3.09 ± 1.77 (− 6.02–0.36)	3.16 ± 1.63 (0.36–6.02)	− 0.331 ± 0.19 (− 0.658–0.041)	0.338 ± 0.175 (0.041–0.658)	− 3.09 ± 1.77 (− 6.02–0.36)	3.16 ± 1.63 (0.36–6.02)	− 0.331 ± 0.19 (− 0.658–0.041)	0.338 ± 0.175 (0.041–0.658)
	Bone	− 1.31 ± 1.98 (− 4.58–2.9)	1.86 ± 1.41 (0.12–4.58)	− 0.146 ± 0.24 (− 0.482–0.427)	0.227 ± 0.156 (0.017–0.482)	− 1.31 ± 1.98 (− 4.58–2.9)	1.86 ± 1.41 (0.12–4.58)	− 0.146 ± 0.24 (− 0.482–0.427)	0.227 ± 0.156 (0.017–0.482)
	Kidneys	− 0.56 ± 1.88 (− 5.31–1.37)	1.2 ± 1.51 (0.07–5.31)	− 0.037 ± 0.132 (− 0.284–0.109)	0.097 ± 0.093 (0.005–0.284)	− 0.56 ± 1.88 (− 5.31–1.37)	1.2 ± 1.51 (0.07–5.31)	− 0.037 ± 0.132 (− 0.284–0.109)	0.097 ± 0.093 (0.005–0.284)
	Spleen	− 0.05 ± 2.3 (− 5.15–3.85)	1.6 ± 1.57 (0.11–5.15)	0.011 ± 0.188 (− 0.337–0.356)	0.138 ± 0.12 (0.009–0.356)	− 0.05 ± 2.3 (− 5.15–3.85)	1.6 ± 1.57 (0.11–5.15)	0.011 ± 0.188 (− 0.337–0.356)	0.138 ± 0.12 (0.009–0.356)
	Bladder	2.39 ± 3.73 (− 2.89–11.64)	3.11 ± 3.1 (0.48–11.64)	0.229 ± 0.328 (− 0.221–1.05)	0.282 ± 0.28 (0.06–1.05)	2.39 ± 3.73 (− 2.89–11.64)	3.11 ± 3.1 (0.48–1.64)	0.229 ± 0.328 (− 0.221–1.05)	0.282 ± 0.28 (0.06–1.05)
	Lungs	− 2.42 ± 1.3 (− 4.34– − 0.45)	2.42 ± 1.3 (0.45–4.34)	− 0.275 ± 0.135 (− 0.473– − 0.046)	0.275 ± 0.135 (0.046–0.473)	− 2.42 ± 1.3 (− 4.34–0.45)	2.42 ± 1.3 (0.45–4.34)	− 0.275 ± 0.135 (− 0.473– − 0.046)	0.275 ± 0.135 (0.046–0.473)
	Brain	− 3.52 ± 3.25 (− 7.87–3.05)	4.15 ± 2.29 (0.43–7.87)	− 0.587 ± 0.525 (− 1.198–0.45)	0.681 ± 0.38 (0.066–1.198)	− 3.52 ± 3.25 (− 7.87–3.05)	4.15 ± 2.29 (0.43–7.87)	− 0.587 ± 0.525 (− 1.198–0.45)	0.681 ± 0.38 (0.066–1.198)
	All Organs	− 1.10 ± 2.89 (− 7.87–11.63)	2.34 ± 2.01 (0.07–11.63)	− 0.144 ± 0.348 (− 1.198–1.049)	0.270 ± 0.261 (0.005–1.198)	− 1.11 ± 2.90 (− 7.84 –11.76)	2.34 ± 2.03 (0.08–11.76)	− 0.144 ± 0.342 (− 1.497–0.765)	0.23 ± 0.28 (0.01–1.49)

**Table 4:** Outcome of organ-wise dose estimates for the 90 kVp dataset after fine-tuning

		FTC				TCM			
	*Organ*	RE %	RAE %	ME (mGy)	MAE (mGy)	RE %	RAE %	ME (mGy)	MAE (mGy)
90 kVp	Liver	− 0.41 ± 1.83 (− 3.3–3.76)	1.37 ± 1.22 (0.14–3.76)	− 0.04 ± 0.155 (− 0.305–0.299)	0.112 ± 0.109 (0.009–0.305)	− 0.5 ± 1.81 (− 3.15–3.78)	1.37 ± 1.22 (0.07–3.78)	− 0.036 ± 0.114 (− 0.262–0.211)	0.08 ± 0.085 (0.001–0.262)
	Heart	− 3.81 ± 2.3 (− 6.29–1.03)	4.01 ± 1.9 (0.04–6.29)	− 0.325 ± 0.207 (− 0.557–0.095)	0.343 ± 0.173 (0.002–0.557)	− 3.85 ± 2.32 (− 6.27–1.03)	4.04 ± 1.92 (0.04–6.27)	− 0.24 ± 0.177 (− 0.455–0.078)	0.255 ± 0.153 (0.002–0.455)
	Bone	− 3.16 ± 2.72 (− 5.72–4.15)	3.92 ± 1.22 (1.56–5.72)	− 0.282 ± 0.255 (− 0.463–0.433)	0.361 ± 0.097 (0.167–0.463)	− 3.06 ± 2.75 (− 5.72–4.2)	3.82 ± 1.34 (0.8–5.72)	− 0.212 ± 0.223 (− 0.491–0.353)	0.276 ± 0.124 (0.101–0.491)
	Kidneys	− 1.81 ± 2.12 (− 6.42–0.27)	1.86 ± 2.07 (0.2–6.42)	− 0.101 ± 0.11 (− 0.33–0.014)	0.104 ± 0.107 (0.01–0.33)	− 2.03 ± 2.08 (− 6.42–0)	2.03 ± 2.08 (0–6.42)	− 0.078 ± 0.08 (− 0.223–0)	0.078 ± 0.08 (0 –0.223)
	Spleen	− 1.33 ± 2.08 (− 4.5–2.05)	1.99 ± 1.39 (0.16–4.5)	− 0.085 ± 0.134 (− 0.314–0.12)	0.127 ± 0.091 (0.007–0.314)	− 1.25 ± 2.22 (− 4.7–2.77)	1.96 ± 1.56 (0.05–4.7)	− 0.056 ± 0.079 (− 0.203–0.083)	0.075 ± 0.059 (0.001–0.203)
	Bladder	1.59 ± 4.3 (− 3.66–11.86)	3.1 ± 3.27 (0.31–1.86)	0.109 ± 0.285 (− 0.275–0.787)	0.211 ± 0.213 (0.013–0.787)	1.61 ± 4.22 (− 3.57–11.74)	3.05 ± 3.23 (0.31–1.74)	0.067 ± 0.133 (− 0.083–0.383)	0.101 ± 0.106 (0.013–0.383)
	Lungs	1.42 ± 2.5 (− 2.35–6.59)	2.12 ± 1.88 (0.11–6.59)	0.112 ± 0.211 (− 0.236–0.549)	0.178 ± 0.154 (0.008–0.549)	1.46 ± 2.43 (− 2.25–6.4)	2.09 ± 1.86 (0.04–6.4)	0.096 ± 0.165 (− 0.133–0.425)	0.14 ± 0.125 (0.002–0.425)
	Brain	2.19 ± 4.88 (− 3.72–14.13)	3.57 ± 3.88 (0.06–14.13)	0.239 ± 0.56 (− 0.522–1.528)	0.426 ± 0.421 (0.008–1.528)	2.11 ± 4.74 (− 3.72–13.57)	3.5 ± 3.73 (0.15–13.57)	0.37 ± 0.884 (− 0.522–2.702)	0.572 ± 0.756 (0.013–2.702)
	All Organs	− 0.67 ± 3.59 (− 6.42–14.13)	2.74 ± 2.40 (0.04–14.13)	− 0.047 ± 0.321 (− 0.557–1.528)	0.233 ± 0.224 (0.002–1.528)	− 0.69 ± 3.57 (− 6.42–13.57)	2.73 ± 2.38 (0.01–13.57)	− 0.011 ± 0.375 (− 0.522–2.702)	0.197 ± 0.318 (0.001–2.701)

## Discussion

In this work, we proposed a novel method for dose map calculation using deep neural networks through two input channels. The model estimates the radiation voxel dose map by combining the attenuation and source angle/position information from the SP_uniform image with the attenuation characteristics from the density map image (Figure 2). This model predicts the dose distribution corresponding to a single source position/angle around the patient's body, which can be an excellent option to calculate the absorbed doses with a lower interval in the source position movements, which proved to be more realistic [30]. Other acquisition parameters, such as pitch, scan mode (spiral, sequential), rotation time, and other parameters, such as tube current, could be modeled by providing single-source position dose maps. This capability of calculating single source position and angle enables the calculation of whole-body dose maps in more complicated acquisition settings, such as organ-based TCM algorithms where the tube current is reduced in anterior arcs. The proposed DNN model generates a single source position/angle from two inputs, namely SP-uniform and density maps. The DNN output (SPDL) is the absorbed dose for a fixed unit of tube current. Then, this SP_DL is multiplied by a value depending on the tube current at this point to model the TCM or FTC scenario. The DNN error is limited to the first step, i.e. the TCM/FTC tube current correction is reproducible and there is no error in this step. This feature makes our model parameter-free and our results confirmed this assertion by showing so close errors for TCM and FTC scenarios. Acquisition parameters may be divided into two subcategories:

i. Parameters affecting the tube output, such as manufacturer/model parameters (inherent beam filtration, anode material, anode angel, high-frequency characteristics,…), kVp, tube current wither as a fixed value (FTC), modulated according to patient body habitus (TCM), or more recent organ-based TCM.
ii. Parameters affecting or depending on the geometry and position of the X-ray interaction with patient’s body, including table height, bowtie filter, pitch factor, rotation time, and patient’s body shape and composition. If a TCM algorithm is used, the tube current at each point depends on the geometry of the tube source with respect to the patient’s body.

Using our proposed methodology, all parameters in the second category can be modeled since they depend on (i) the position, angle, and distance of the source to patient’s body and (ii) patient’s body shape and composition. Our model can extract the related information for model “i” from the SP-uniform image and “ii” from the patient density map. Among the above-mentioned parameters, the only parameter which affects the beam energy spectrum (for a single scanner) is tube voltage. We attempted to fine-tune our trained network on 120 kVp using another kVp (90 kVp) to demonstrate that this limitation may be overcome with less effort through transfer learning. In other words, the proposed model is a parameter-free protocol except for the scanner manufacturer/model and kVp, where the same results can be achieved through fine-tuning. It should be emphasized that MC simulations are necessary for fine-tuning (training again) for a new kVp or scanner/model. Conversely, after fine-tuning or when using the model for the same kVp and scanner/model, the inference step does not require MC calculations.

The model’s generalizability was examined through transfer learning to a different kVp dataset and fine-tuning the model. The results in terms of organ-wise dose metrics demonstrated the robustness of the developed model. Our model’s performance was similar when considering FTC and TCM techniques in both voxel-wise and organ-wise metrics. MC calculations take between 2 and 3 days for a total body simulation depending on patient’s size. While our proposed methodology can be performed within few minutes taking advantage of fast parallel processing on GPUs, this computation time is acceptable for achieving a dose map comparable with MC calculations in terms of accuracy. Besides, the dose map calculation is feasible for an acquisition performed using dual-source CT scanners or single-source scanners operating in dual-energy mode by considering the source from each kVp (X-ray tube) as a single source position/angle. However, owing to the different energy ranges and detector technology in new photon-counting CT detectors, additional fine-tuning cases or different strategies for fine-tuning might be needed. Caution is commended when using fine-tuning strategy in this particular case.

The patients included in the training and test datasets covered a wide range of body shapes and BMIs. As shown in Figure 4, the model is robust against patient size and composition changes. Wang et al [35] proposed analytic linear Boltzmann modeling of the radiation dose in an anthropometric phantom. They reported errors of less than 3%, but their model was specific for a single phantom and didn't consider variability in the human body. Although performed independently, our study bears some similarities with the study published by Maier et al [25] in the sense that we used two channel inputs to our model to predict the voxel dose maps. We used whole-body CT images covering a larger axial field-of-view range from the skull to mid-thigh and trained a single general model for the full coverage. The single general model applicable to all scan protocols is easier to implement. They introduced multiple models by changing the parameters, while the generalizability is more practical in daily clinical routine by accessing single source dose maps. Besides, our proposed methodology is capable of reconstructing the dose maps directly from CT images without additional time-consuming deterministic methods for solving the Boltzmann transport equation.

Tzanis et al [26] used DL to generate voxel dose maps calculated by Monte Carlo simulations by converting the image into a long vector and introducing scan parameters, such as tube current and scan mode, as additional columns. They included 343 head and neck scans and reported organ doses delivered to three tissues/organs, including the brain, cranial bones, and eye lens, with average errors less than 6% (range 0–19%) in terms of organ RAE. Our proposed model provides more accurate results in terms of organ-wise RAE (average 2.74). Besides, they only used 120 kVp acquisitions and a scan range limited to the head and neck region.

Organ masks are a critical requirement for calculating organ doses. We segmented multiple organs to evaluate the performance of our model in organ-level dose calculation. The labor-intensive and time-consuming segmentations are important limitations of using dose maps in radiation risk estimation. Despite the presence of voxels with a higher error than the average in terms of voxel-wise RAE (%), the organ dose errors were negligible, especially for large organs, such as the liver. The slightly higher error in some organ doses and voxels could be attributed to methodological limitations, such as coarse image matrix size (voxel size of 5 mm), that we adopted to reduce the computational time. The large voxel size can also cause higher errors in voxel-wise metrics. The excellent performance achieved by our model in organ-level doses is much better than pre-tabulated software outputs. Moreover, we used only a single scanner to train our DL network using a limited number of patients. Although we proved the validity of the concept of transfer learning for other X-ray spectra with comparable error and robust results on the same scanner, it should be noted that each scanner, even the same model/manufacturer and has a specific output energy spectra that should be considered during fine-tuning. Still, the main bottleneck was the high computational time required to generate the Monte Carlo dose maps as ground truth. In addition, in our method, the radiation dose delivered to organs out of the scan reconstruction axial range is missing.

## Conclusion

Our proposed deep learning model can generate whole-body dose maps from a CT scan acquisition with reasonable accuracy at the voxel level and excellent performance at organ-level dose estimation. The whole process, including pre-processing and model inference on a new dataset, can be performed within seconds, which makes personalized dosimetry with an acceptable accuracy a possible option in clinical routine. Conversely, by generating a dose distribution from a single source position, our model can generate accurate and personalized dose maps for a wide range of acquisition parameters.

### Supplementary Information

Below is the link to the electronic supplementary material.

Supplementary file1 (PDF 155 KB)

## Abbreviations

CT: Computed tomography
DL: Deep learning
ICRP: International Commission on Radiological Protection
MAE: Mean absolute error
MC: Monte Carlo
ME: Mean error
PSNR: Peak signal-to-noise ratio
RAE: Relative absolute error
RE: Relative error
SP_DL: Single-point Deep learning dose
SP_MC: Single-point Monte Carlo dose
SP_uniform: Single-point Monte Carlo dose in a uniform material
SSIM: Structural similarity index
TCM: Tube current modulation
WBCT: Whole-body computed tomography
WBDM: Whole-body dose map

## Footnotes

1 https://impactdose.software.informer.com/.

## Declarations

### Guarantor

The scientific guarantor of this publication is Habib Zaidi.

### Statistics and biometry

No complex statistical methods were necessary for this paper.

### Study subjects or cohorts overlap

Study subjects or cohorts have not been previously reported.

### Methodology

• retrospective

• experimental

• mono-centric, single-institution
