# Supplementary material for: Real-time, acquisition parameter-free voxel-wise patient-specific Monte Carlo dose reconstruction in whole-body CT scanning using deep neural networks
Source: Eur Radiol. 2023 Jun 27;33(12):9411–24. doi: 10.1007/s00330-023-09839-y (PMC10667156; doi:10.1007/s00330-023-09839-y)
Supplement: Supplementary file 1 — Supplementary file1 (PDF 155 KB) [file 330_2023_9839_MOESM1_ESM.pdf]

This figure shows an example of SP_uniform image cropped to body contour segmentation while the source is rotation in clockwise direction from the top left image to the bottom right image

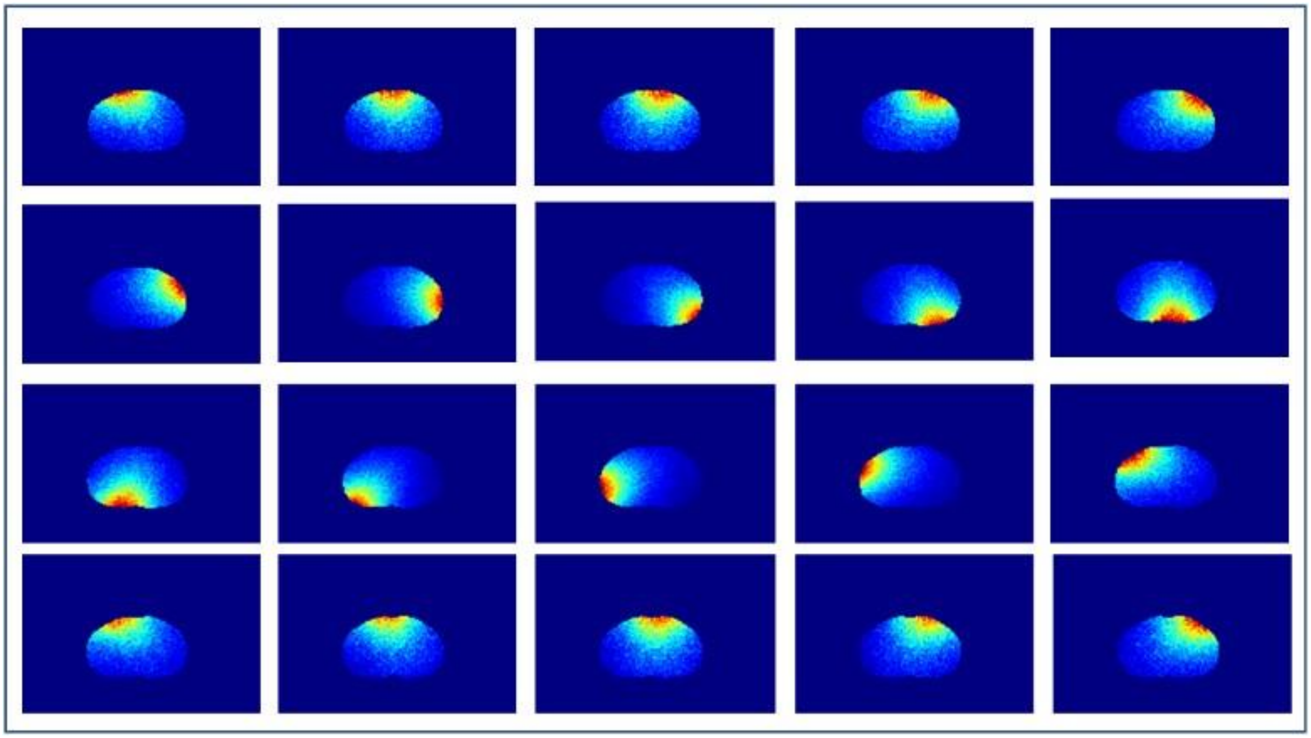

Figure 1 - supplementary. The rotating source around the patient's body and the resulting SP-uniform image

Eur Radiol (2023) Salimi Y, Akhavanallaf A, Mansouri Z, Shiri I, Zaidi H
